# Case Report: A Rare Case of Benign Recurrent Intrahepatic Cholestasis-Type 1 With a Novel Heterozygous Pathogenic Variant of *ATP8B1*

**DOI:** 10.3389/fmed.2022.891659

**Published:** 2022-04-29

**Authors:** Hiroyuki Suzuki, Teruko Arinaga-Hino, Tomoya Sano, Yutaro Mihara, Hironori Kusano, Tatsuki Mizuochi, Takao Togawa, Shogo Ito, Tatsuya Ide, Reiichiro Kuwahara, Keisuke Amano, Toshihiro Kawaguchi, Hirohisa Yano, Masayoshi Kage, Hironori Koga, Takuji Torimura

**Affiliations:** ^1^Division of Gastroenterology, Department of Medicine, Kurume University School of Medicine, Kurume, Japan; ^2^Department of Pathology, Kurume University School of Medicine, Kurume, Japan; ^3^Department of Diagnostic Pathology, National Hospital Organization Kokura Medical Center, Fukuoka, Japan; ^4^Department of Pediatrics and Child Health, Kurume University School of Medicine, Kurume, Japan; ^5^Department of Pediatrics and Neonatology, Nagoya City University Graduate School of Medical Sciences, Nagoya, Japan; ^6^Department of Medical Engineering, Junshin Gakuen University, Fukuoka, Japan

**Keywords:** benign recurrent intrahepatic cholestasis (BRIC), *ATP8B1*, autosomal recessive, cholestasis, progressive familial intrahepatic cholestasis (PFIC), rifampicin

## Abstract

Benign recurrent intrahepatic cholestasis type 1 (BRIC1) is a rare autosomal recessive disorder that is characterized by intermittent episodes of jaundice and intense pruritus and caused by pathogenic variants of *adenosine triphosphatase phospholipid transporting 8B1* (*ATP8B1*). The presence of genetic heterogeneity in the variants of *ATP8B1* is suggested. Herein, we describe a unique clinical course in a patient with BRIC1 and a novel heterozygous pathogenic variant of *ATP8B1*. A 20-year-old Japanese man experienced his first cholestasis attack secondary to elevated transaminase at 17 years of age. Laboratory examinations showed no evidence of liver injury caused by viral, autoimmune, or inborn or acquired metabolic etiologies. Since the patient also had elevated transaminase and hypoalbuminemia, he was treated with ursodeoxycholic acid and prednisolone. However, these treatments did not relieve his symptoms. Histopathological assessment revealed marked cholestasis in the hepatocytes, Kupffer cells, and bile canaliculi, as well as a well-preserved intralobular bile duct arrangement and strongly expressed bile salt export pump at the canalicular membrane. Targeted next-generation sequencing detected a novel heterozygous pathogenic variant of *ATP8B1* (c.1429 + 2T > G). Taken together, the patient was highly suspected of having BRIC1. Ultimately, treatment with 450 mg/day of rifampicin rapidly relieved his symptoms and shortened the symptomatic period.

## Introduction

Pathogenic variants in the *adenosine triphosphatase phospholipid transporting 8B1 (ATP8B1)* gene cause progressive familial intrahepatic cholestasis type 1 (PFIC1) and benign recurrent intrahepatic cholestasis type 1 (BRIC1) (1). The former is a progressive disease involving persistent cholestasis that eventually leads to liver cirrhosis, while the latter has a benign clinical course with recurrent cholestasis attacks and asymptomatic periods (1). *ATP8B1* affects the regulation of the Farnesoid X receptor, and a deficiency of *ATP8B1* protein may cause an imbalance between intestinal absorption and hepatic secretion of bile acids, leading to bile acid accumulation (2). The first cholestasis attack in patients with BRIC1 usually occurs before the second decade of life. Thereafter, several similar attacks with asymptomatic intervals ranging from several months to years may occur. During the asymptomatic intervals in these patients, biochemistry data remain normal. Each attack may last for several weeks to months without treatment, and most cases of BRIC1 do not progress to cirrhosis or end-stage liver disease. Classically, during the attacks in these patients, serum bile salt concentrations and bilirubin values are markedly elevated, whereas gamma-glutamyl transpeptidase (GGT) levels and aminotransferase activities remain within the normal range or are slightly elevated (1). To the best of our knowledge, this is the first report of a BRIC1 patient with a novel heterozygous variant of *ATP8B1*. Herein, we describe a unique clinical course of BRIC1 that deviates from the classical one.

## Case Report

**FIGURE 1:**
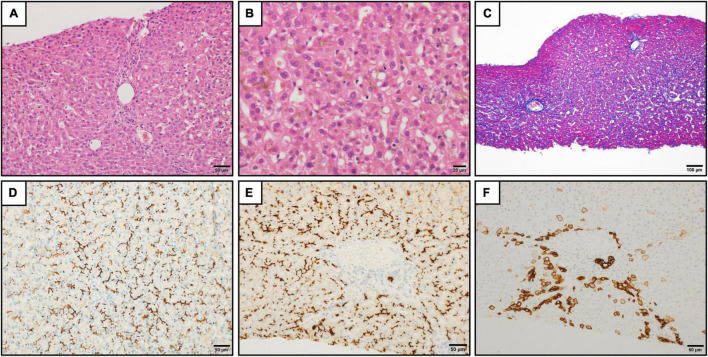
Histopathologic findings in the liver. **(A)** Liver tissue showed no significant lobular architecture (hematoxylin and eosin staining). Scale bar represents 50 μm. **(B)** Cholestasis was observed in the hepatocytes, Kupffer cells, and bile canaliculi at zones 2 and 3 (hematoxylin and eosin staining). Scale bar represents 20 μm. **(C)** There was no significant fibrosis (Azan staining). Scale bar represents 100 μm. **(D–F)** Immunohistochemistry for bile salt export pump **(D)**, CD10 **(E)**, and keratin 7 **(F)**. The strong expression of bile salt export pump and CD10 were observed at the canalicular membrane **(D,E)**. The keratin 7 expression was found in the hepatocytes in the periportal areas. The intralobular bile duct arrangement was preserved **(F)**. Scale bars represent 50 μm.

**FIGURE 2:**
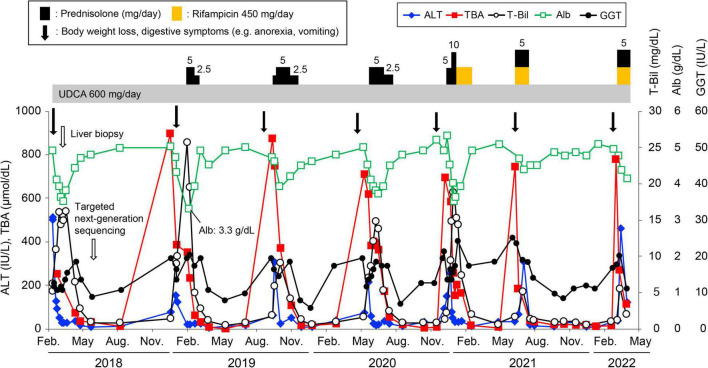
Clinical course of the patient. Changes in serum alanine aminotransferase (ALT, ◆), total bile acid (TBA, ■), total bilirubin (T-Bil, ○), albumin (Alb, □), and gamma-glutamyl transpeptidase (GGT, •) levels. The patient was treated with prednisolone or rifampicin with ursodeoxycholic acid during the attacks. The lowest albumin level during the follow-up period was 3.3 g/dL.

A 20-year-old Japanese man experienced his first cholestasis attack at the age of 17 years old with the following laboratory findings; serum total bilirubin, 16.0 mg/dL; alkaline phosphatase (ALP), 682 IU/L; GGT activity, 16 IU/L; serum aspartate aminotransferase (AST), 158 IU/L; and alanine aminotransferase (ALT), 518 IU/L (Table 1). The patient had no risk factors for liver injury, such as drug use, toxin ingestion, alcohol abuse, and inborn or acquired metabolic etiologies. Parameters suggestive of autoimmune hepatitis or viral hepatitis, including antinuclear antibodies, antimitochondrial antibody, hepatitis B surface antigen, hepatitis C virus antibody, cytomegalovirus antibody, and Epstein-Barr virus, were negative (Table 1). Magnetic resonance cholangiopancreatography showed a normal intrahepatic and extrahepatic biliary tree and pancreatic ductal system. The liver tissue obtained from a percutaneous liver biopsy showed marked cholestasis in the hepatocytes, Kupffer cells, and bile canaliculi; however, no lobular inflammation, hepatic necrosis, or fibrosis were found (Figures 1A–C). He was treated with ursodeoxycholic acid (UDCA) at a dose of 600 mg/day, and its intermittent effect was obtained at the initial stage. The patient was then referred to our hospital for further examination and treatment. Serological parameters of liver fibrosis had the following values: serum type IV collagen, 237.0 ng/mL and Mac-2 binding protein glycosylation isomer, 0.23 (Table 1). Liver stiffness measured by transient elastography (FibroScan^®^) was 7.8 kPa. Further pathological assessment and immunohistochemistry studies revealed that bile salt export pump, multidrug resistance protein 3, multidrug resistance-associated protein 2, and CD10 were strongly expressed at the canalicular membrane (Figures 1D–E) and that the intralobular bile duct arrangement was well-preserved (Figure 1F). After obtaining informed consent, a genetic examination was performed to confirm the clinical diagnosis. Targeted next-generation sequencing, involving 61 genes responsible for genetic disorders of hepatic cholestasis (3), detected a heterozygous variant of *ATP8B1*: c.1429 + 2T > G (NM_005603.4). Thus the patient was highly suspected to have BRIC1 owing to these clinical, genetic, and pathological findings. Thereafter, similar attacks recurred with 5–6 months of asymptomatic intervals. During each attack, the patient experienced strong digestive symptoms associated with >5 kg of body weight loss and hypoalbuminemia (Figure 2). In addition, he experienced severe anorexia and frequent vomiting. There was no trace of inflammation, such as fever or an elevated C-reactive protein level, and esophagogastroduodenoscopy showed only Los Angeles grade B reflux esophagitis. Since every attack was accompanied by rapid transaminase elevation, the patient was treated with 2.5–10 mg/day of prednisolone; however, symptoms such as jaundice, pruritus, and weight loss lasted for 2–3 months. After approval by our hospital ethics committee, the patient was treated with 450 mg/day of rifampicin, which was based on previous studies (4, 5). This treatment rapidly relieved his symptoms and markedly shortened the symptomatic phase compared to prednisolone (Figure 2). Rifampicin was discontinued after the biochemical data improved to normal values. With 6–9 months of asymptomatic intervals, he experienced another two similar attacks. Since each attack was also accompanied by severe anorexia and elevated aminotransferases, we added rifampicin and prednisolone on UDCA. Thereafter, the symptomatic phase was markedly shortened (Figure 2).

**TABLE 1:** Laboratory data on admission at previous hospital.

Biochemistry		Reference range	Peripheral blood		Reference range
TP	7.9 g/dL	6.7–8.3	WBC	6,020/μL	4,000–8,000
Albumin	4.9 g/dL	4.0–5.0	Neutrophil	56.7%	45.0–55.0
T-Bil	16.0 mg/dL	0.3–1.2	Lymphocyte	24.6%	24.0–45.0
D-Bil	8.5 mg/dL	0.0–0.4	Monocyte	10.1%	4.0–7.0
AST	158 IU/L	13–33	Eosinophil	6.1%	1.0–5.0
ALT	518 IU/L	6–30	Basophil	2.5%	0–2.0
LDH	222 IU/L	119–229	Atypical lymphocyte	0%	0
ALP	682 IU/L	115–359	RBC	490 × 10^4^/μL	435 × 10^4^–555 × 10^4^
γ-GTP	16 IU/L	10–47	Hb	14.8 g/dL	13.7–16.8
BUN	12.4 mg/dL	8.0–22.0	Ht	43.7%	35.0–52.0
Cre	0.53 mg/dL	0.60–1.10	Plt	22.5 × 10^4^/μL	12.0 × 10^4^–40.0 × 10^4^
			
Na	141 mEq/L	138–146	**Coagulation**		
			
K	4.0 mEq/L	3.6–4.9	PT%	103.9%	80–120
Cl	105 mEq/L	99–109	PT-INR	1.01	0.84–1.14
			
CRP	0.03 mg/dL	0.0–0.3	**Serology**		
			
TBA	253.4 μmol/dL	≤14.4	ANA	(−)	
TSH	0.3 μIU/L	0.4–4.0	AMA/AMA-M2	(−)/(−)	
FT4	1.03 ng/dL	0.8–1.9	M2BPGi*	(−)	
IgA	217 mg/dL	90–400	Type IV collagen*	237.0 ng/mL	142–600
			
IgM	85 mg/dL	31–200	**Viral markers**		
			
IgG	1,108 mg/dL	820–1740	IgM anti-HAV	(−)	
IgG4	24.9 mg/dL	≤135	HBsAg	(−)	
Ferritin	1,153.5 ng/dL	21–282	Anti-HBc	(−)	
Cu	146 μg/dL	66–130	Anti-HCV	(−)	
Ceruloplasmin	31.8 mg/dL	21.0–37.0	IgA anti-HEV	(−)	
Fischer’s ratio	2.40	2.43–4.40	IgM/IgG anti-CMV	(−)/(−)	
			IgM/IgG anti-EB-VCA	(−)/(+)	
			IgG anti-EBNA	(+)	
			IgM/IgG anti-Parvovirus B19	(−)/(+)	
			Anti-HTLV-1	(−)	

*TP, total protein; T-Bil, total bilirubin; D-Bil, direct bilirubin; AST, aspartate aminotransferase; ALT, alanine aminotransferase; LDH, lactate dehydrogenase; ALP, alkaline phosphatase; γ-GTP, γ-glutamyl transpeptidase; BUN, blood urea nitrogen; Cre, creatinine; CRP, C-reactive protein; TBA, total bile acid; TSH, tyrosine stimulating hormone; FT4, Free T4; WBC, white blood cell; RBC, red blood cell; Hb, hemoglobin; Ht, Hematocrit; Plt, Platelet count; PT, prothrombin time; INR, international normalized ratio; ANA, antinuclear antibody; AMA, anti-mitochondria antibody; AMA-M2, antimitochondrial M2 antibody; M2BPGi, Mac-2 binding protein glycosylation isomer; HAV, hepatitis A virus; Ag, antigen; HBV, hepatitis B virus; HCV, hepatitis C virus; HEV: hepatitis E virus; CMV, cytomegalovirus; EB-VCA, Epstein-Barr viral capsid antigen; EBNA, Epstein-Barr nuclear antigen; HTLV-1, human T-cell leukemia virus type 1.*

*These parameters were obtained at our hospital.

## Discussion

BRIC1 is a very rare autosomal recessive genetic disorder that was first described by Summerskill and Walshe in 1959. This condition is characterized by recurrent episodes of jaundice and intense pruritus, classically without the development of severe hepatic fibrosis (6). Since its discovery, many reports have been published, but the pathogenesis of BRIC1 remains unclear. Recently, molecular and genetic analyses have demonstrated that BRIC1 is caused by pathogenic variants in *ATP8B1* at chromosome 18q21. In patients with *ATP8B1* deficiency, the asymmetry of aminophospholipids in the hepatocanalicular membrane is disrupted, and the transport activity of the bile acid transport pump, which is an ABC transporter localized on the hepatocanalicular membrane that mediates the excretion of bile acids, is reduced, thus resulting in the development of severe intrahepatic cholestasis (7, 8). Moreover, because only a few bile acids flow into the bile canaliculus, it is considered that the cholangiocytes existing in the bile canaliculus and intrahepatic and extrahepatic biliary tree are not damaged, which could explain the normal GGT activity in these patients (7, 8). In the current case, the molecular genetic analysis revealed that the patient had a heterozygous variant of *ATP8B1* with c.1429 + 2T > G. According to the guideline for determining the pathogenicity of identified variants (9), we judged the variant as a novel pathogenic variant, because this was not registered in several public databases, such as the Human Gene Mutation Database, Human Genetic Variation Database, and The Genome Aggregation Database (10) and because computational predictive programs strongly suggested its pathogenicity. In addition, we did not identify any pathogenic variant of 61 candidate genes by next-generation sequencing (3). Monoallelic pathogenic variants of *ATP8B1* have been reported to be a predisposing factor for phenotypes such as drug-induced cholestasis, intrahepatic cholestasis of pregnancy type 1, and transient neonatal cholestasis (11); therefore, it is suggested that there may be undiscovered pathogenic variants of *ATP8B1* in patients with BRIC1. Although the patient in this case had a variant of *ATP8B1* in the heterozygous state, he was highly suspected of BRIC1 because of his clinical, genetic, and pathological findings. To the best of our knowledge, this is the first report regarding this novel pathogenic variant of *ATP8B1* in a patient with BRIC1.

Although classical BRIC1 does not progress to cirrhosis or end-stage liver disease, a previous study has reported that four cases of BRIC1 which showed progression to a more severe and permanent form of PFIC (12). This indicates the possibility of clinical continuity with an intermediate phenotype between BRIC1 and PFIC1. The clinical course in the patient in the current case differed from classical BRIC1 in that it was accompanied by very strong gastrointestinal symptoms, elevated transaminases, and hypoalbuminemia, suggesting that it may be an intermediate phenotype of these diseases. Since the phenotype in this case deviates from that in classical BRIC1, we conducted general examinations, including the evaluation of Epstein-Barr virus, cytomegalovirus, and parvovirus B19, to investigate the cause of rapid transaminase elevation. Although no obvious causes were detected, the possibility of unknown causes (including another viral infections) triggering cholestasis cannot be ruled out. In addition, this difference may also be due to the rare genetic variant of *ATP8B1*. In a recent Japanese nationwide survey, it was shown that there are no critical methods to distinguish between PFIC1 and BRIC1 at the early phase of the disease (13). Therefore, further accumulation and evaluation of BRIC1 cases are desirable for more appropriate follow-up of these patients.

The effects of several therapeutic strategies aimed at relieving symptoms during cholestasis attacks in patients with BRIC1, such as UDCA, corticosteroids, cholestyramine, and nasobiliary drainage, are known to be case-dependent and usually ineffective (14, 15). In this report, since the patient had elevated aminotransferases, we added prednisolone to UDCA. However, these two drugs did not seem to be effective. Treatment with rifampicin, a potent human activator of pregnane X receptor, improves pruritus, shortens the symptomatic phase, and extends the asymptomatic interval (5). Consistent with previous reports, treatment with rifampicin for the patient in the current case markedly shortened the symptom duration (4, 16). In contrast, little benefit from rifampicin regarding extension of the asymptomatic interval was obtained. It has also been reported that the long-term administration of rifampicin may result in severe hepatoxicity in patients with cholestatic disorders (16). Therefore, it is also important the timing of drug discontinuation, which needs to be further evaluated.

## Conclusion

We reported a rare case of a young Japanese patient with BRIC1 and a novel pathogenic variant of *ATP8B1*. We found that his phenotype was different from those of classical BRIC1 in that it was accompanied by rapid transaminase elevation. In addition, the little benefit from prednisolone and the effectiveness of rifampicin were obtained.

## Data Availability Statement

The original contributions presented in the study are included in the article/supplementary material, further inquiries can be directed to the corresponding author.

## Ethics Statement

Ethical review and approval was not required for the study on human participants in accordance with the local legislation and institutional requirements. The patients/participants provided their written informed consent to participate in this study.

## Author Contributions

HS and TA-H wrote the manuscript. HKu, HY, KA, HKo, MK, and TTr supervised the study. YM, TM, TI, RK, KA, TK, and TS participated in the acquisition of data and critical revision. TTg and SI performed genetic analysis and revised the manuscript. All authors discussed the results and contributed to the final manuscript.

## Conflict of Interest

The authors declare that the research was conducted in the absence of any commercial or financial relationships that could be construed as a potential conflict of interest.

## Publisher’s Note

All claims expressed in this article are solely those of the authors and do not necessarily represent those of their affiliated organizations, or those of the publisher, the editors and the reviewers. Any product that may be evaluated in this article, or claim that may be made by its manufacturer, is not guaranteed or endorsed by the publisher.
